# Clinical efficacy of CO_2_ fractional laser combined with compound betamethasone in treating vitiligo and its impact on inflammatory factors

**DOI:** 10.3389/fmed.2024.1408409

**Published:** 2024-07-10

**Authors:** Lina Zhang, Jianzhong Zhang, Xin Wang, Zhonglin Zhao, Zhifeng Li, Guoying Miao, Chao Lv

**Affiliations:** Dermatology Department, Affiliated Hospital of Hebei Engineering University, Handan, China

**Keywords:** CO_2_ fractional laser, compound betamethasone, vitiligo, clinical efficacy, inflammatory factors, impact

## Abstract

**Objective:**

To analyze the clinical efficacy of CO_2_ fractional laser combined with compound betamethasone in treating vitiligo and its impact on inflammatory factors.

**Methods:**

The clinical treatment effects, levels of inflammatory factors [interleukin-17 (IL-17), interferon-gamma (IFN-γ), interleukin-10 (IL-10)], prognosis regarding repigmentation and relapse, psychological health (satisfaction).

**Results:**

① Clinical treatment effects: the total effective rate in Group A was 92.73%, Group B was 74.55%, and Group C was 67.27%, with Group A showing significantly higher effectiveness than Groups B and C (*p* < 0.05). ② Inflammatory factors: prior to treatment, there was no significant difference in IL-17, IFN-γ, and IL-10 levels among the three groups (*p* > 0.05); after 3 and 6 months of treatment, the levels of IL-17 and IFN-γ decreased significantly while IL-10 levels increased significantly across all three groups, with Group A showing a more pronounced change compared to Groups B and C (*p* < 0.05). ③ Prognosis regarding repigmentation and relapse: after 3 and 6 months of treatment, Group A exhibited significantly higher repigmentation rates compared to Groups B and C (*p* < 0.05); in terms of relapse, Group A had a relapse rate of 5.45%, Group B had 21.82%, and Group C had 23.64%, with Group A showing significantly lower relapse rates compared to Groups B and C (*p* < 0.05). ④ Quality of life and psychological health: at the end of the 6 month follow-up, the quality of life and psychological health of patients in Group A were significantly higher than those in Groups B and C (*p* < 0.05). ⑤ Occurrence of adverse reactions: the incidence of adverse reactions was 12.73% in Group A, 10.91% in Group B, and 9.09% in Group C, with no significant difference observed among the three groups (*p* > 0.05).

**Conclusion:**

The application of CO_2_ fractional laser combined with compound betamethasone in vitiligo patients demonstrates significant efficacy. Compared to sole treatment with CO_2_ fractional laser or compound betamethasone injections, this combined approach further improves the levels of inflammatory factors in vitiligo patients, reduces the risk of relapse, enhances skin repigmentation, improves quality of life, psychological well-being, without increasing the risk of related adverse reactions. This combined approach merits clinical promotion and application.

## 1 Introduction

Vitiligo is a commonly encountered pigmentary disorder clinically characterized by localized or generalized depigmentation or hypopigmented patches on the skin and mucous membranes. It is easily diagnosed yet challenging to treat, often presenting a high recurrence rate and poor prognosis, significantly impacting the quality of life and psychological well-being of affected individuals (1). Traditional treatment approaches for vitiligo include oral and topical corticosteroids, immunomodulatory therapies, phototherapy, and epidermal grafting. Due to the unclear pathogenesis of vitiligo and the unsatisfactory treatment outcomes, the inefficiency of single-method therapies and their prolonged treatment periods, there exists an urgent need to explore novel treatment modalities for vitiligo (2). Carbon dioxide (CO_2_) fractional laser is a minimally invasive cosmetic dermatological technology used in the treatment of various skin conditions, including vitiligo. It works by emitting a laser beam that penetrates the skin, creating microscopic channels known as microthermal zones (MTZs) while leaving the surrounding tissue unaffected. This fractional approach allows for targeted treatment of specific areas, promoting skin regeneration and pigment restoration (3). It disrupts the pigment balance between normal skin and vitiliginous lesions, promoting the migration and proliferation of melanocytes towards the lesions, stimulating collagen activity, and accelerating rapid wound repair. Consequently, it has emerged as a new focal point in treating vitiligo (4). Compound betamethasone injections, as a corticosteroid compound, when used in vitiligo patients, maintain long-term efficacy and control clinical symptoms (5). Injection administration allows for more precise delivery of the medication directly to the affected areas, ensuring deeper penetration and potentially more effective modulation of the inflammatory response. Additionally, injections can provide a controlled and sustained release of the corticosteroid, which may enhance therapeutic outcomes compared to topical applications that may not penetrate as deeply. However, there is limited research on the combined use and their clinical effects in treating vitiligo and improving prognosis.

Recent studies (6) have demonstrated the close association between immune cell cytokines mediated inflammatory responses and the development of vitiligo. Reports (7) have indicated a significant elevation of IL-17 and IFN-γ levels in the serum of vitiligo patients, which significantly decrease post-treatment, while IL-10 is markedly reduced in the serum, displaying immune suppression. To date, clinical reports on whether CO_2_ fractional laser combined with compound betamethasone injections in vitiligo treatment modulates IL-17, IFN-γ, and IL-10 inflammatory factors are scarce. Therefore, this project aims to evaluate the clinical efficacy and prognosis of CO_2_ fractional laser combined with compound betamethasone injections in treating vitiligo, elucidating their modulation of the inflammatory response via IL-17, IFN-γ, and IL-10, and their role in vitiligo treatment. The outcomes of this study will provide a theoretical basis for understanding the pathogenesis of vitiligo, introduce new perspectives for vitiligo patient treatment, and hold significant importance in improving patient prognosis and quality of life.

## 2 Objectives and methods

### 2.1 Study subjects

A total of 165 vitiligo patients receiving treatment in our dermatology department from January 2022 to December 2022 were selected as study subjects. Basic data including gender, age, duration of illness, affected sites, and family history of vitiligo were collected. Inclusion criteria: ① all patients met the relevant diagnostic criteria for stable vitiligo outlined in the Vitiligo Diagnosis and Treatment Consensus (8); ② no corticosteroid or phototherapy treatments were administered in the last three months; ③ selected patients provided informed consent, were willing to complete the treatment as required, and agreed to follow-up assessments. Exclusion criteria: ① patients with other skin diseases, infections, or sun allergies; ② pregnant or lactating women; ③ individuals with severe heart, liver, or kidney dysfunction; ④ those with compromised immune function or undergoing prolonged immunosuppressive therapy; ⑤ individuals allergic to phototherapy. Using a random number method, stable vitiligo patients were divided into Group A (*n* = 55), Group B (*n* = 55), and Group C (*n* = 55). Group A received treatment with CO_2_ fractional laser combined with compound betamethasone injections; Group B received only CO_2_ fractional laser treatment; Group C received only compound betamethasone injections.

### 2.2 Methods

Group A received treatment with CO_2_ fractional laser combined with compound betamethasone injections; Group B received only CO_2_ fractional laser treatment; Group C received only compound betamethasone injections.

The CO_2_ fractional laser treatment involved adjusting the treatment device parameters based on the color of the patient’s vitiligo lesions. The treatment employed a full-layer mode with superficial energy of 100–120 mJ/cm^2^ and a coverage of 40%; deep energy of 9–12 mJ/cm^2^ and a coverage of 5%. Following treatment, compound betamethasone injections were topically applied and covered for 4–6 h before removal.

In group C, the compound betamethasone injections were administered via intralesional injections directly into the depigmented lesions. This method was chosen to bypass the normal skin barrier and deliver the medication directly to the target areas, ensuring effective penetration and therapeutic impact on the affected tissues. The injections were carefully administered to ensure that the medication reached the deeper layers of the skin, which might not be as effectively achieved with topical applications. All lesions were treated once, and severe areas were treated twice. Treatment sessions were conducted monthly, with photographic documentation of lesion conditions at each follow-up visit. Monthly follow-ups were conducted for 6 months after treatment completion.

### 2.3 Observational indicators

1. Clinical treatment efficacy: Healing: complete disappearance of depigmented patches, restoration of normal skin color; marked improvement: partial disappearance or reduction of depigmented patches, restoration of normal skin color covering ≥65% of the lesion area; improvement: partial disappearance or reduction of depigmented patches, with the patches restored to normal color covering ≥20% of the lesion area; ineffective: no improvement in patches, pigment regeneration, or enlargement of the patches. Total effective rate = (Healing + Marked improvement + Improvement) cases/total cases × 100%.
2. Levels of inflammatory factor indicators: Before treatment, and at 3 and 6 months post-treatment, 10 mL of fasting venous blood was drawn into vacuum tubes. Samples were centrifuged at 3000 r/min using a refrigerated centrifuge to separate the serum, which was stored at −80°C for subsequent analysis. Enzyme-linked immunosorbent assay (ELISA) was utilized to measure the levels of Interleukin-17 (IL-17), Interferon-gamma (IFN-γ), and Interleukin-10 (IL-10) in the patient’s serum, following the manufacturer’s instructions.
3. Prognosis in terms of re-pigmentation and recurrence: Follow-up at 3 and 6 months post-treatment to record the re-pigmentation and recurrence status of the patients. Re-pigmentation rate = area of restored normal skin/area of damaged skin before treatment × 100%.
4. Quality of life: At the end of the 6 month follow-up, the dermatology life quality index (DLQI) (9) was used to assess the patient’s quality of life. This index consists of 10 questions, with scores ranging from 0–3 for each question, totaling 30 points. The final result was uniformly converted to a percentage, with a score closer to 100 indicating a higher quality of life.
5. Psychological health (satisfaction): At the end of the 6 month follow-up, a self-made “Satisfaction Survey” developed by our hospital was provided to the patients and their families for scoring. This questionnaire consists of 20 questions, each scored out of 5, totaling 100 points. The patient’s satisfaction correlates positively with their psychological health, indicating that a higher satisfaction score reflects better psychological well-being.
6. Occurrence of adverse reactions: Adverse reactions observed in this study included fatigue, burning sensation, blisters, erythema, etc. The occurrence of the above adverse reactions was uniformly recorded by relevant medical staff in our hospital.
7. Immunohistochemical (IHC) analysis: To further understand the biological mechanisms underlying the treatment efficacy, skin biopsies were performed on a subset of patients before and after treatment. These biopsies were used to assess histopathological changes and to perform IHC analysis for markers such as melanocyte presence (using markers like Melan-A and HMB-45), inflammatory markers (such as CD3, CD4, CD8), and other relevant cytokines. The IHC analysis aimed to provide insights into the changes at the cellular level that contribute to the clinical outcomes observed.
8. Pearson correlation analysis: Pearson correlation analysis was conducted to assess the linear relationship between the treatment response and the following variables: family history, gender, and age. Pearson correlation coefficient (*r*) was used to evaluate the correlation between the continuous variable (age) and the treatment response. Additionally, point-biserial correlation coefficient (phi coefficient) was utilized to assess the correlation between two binary variables (family history, gender) and the treatment response.

### 2.4 Statistical analysis

GraphPad Prism 8 was used for graphing, and SPSS 22.0 was utilized for data analysis. For quantitative data, mean and standard deviation were used to describe the distribution, and statistical analyses were conducted using *t*-tests or analysis of variance (ANOVA). For categorical data, frequency and percentage were used to describe the distribution, and statistical analyses were performed using chi-square tests or Fisher’s exact tests. Pearson correlation analysis was conducted to assess the linear relationship between the treatment response and the following variables: family history, gender, and age. A significance level of *p* < 0.05 was considered statistically significant.

## 3 Results

### 3.1 Comparison of basic data

The basic data among the three groups of patients were comparable, showing no significant differences in comparison (*p* > 0.05). Refer to Table 1 for details.

**Table 1:** Comparison of basic data.

	Group A (*n* = 55)	Group B (*n* = 55)	Group C (*n* = 55)	*F*	*p*
Gender				–	–
Male	30	27	28		
Female	25	28	27		
Age (years)	35.76 ± 7.39	35.87 ± 7.43	35.82 ± 7.45	0.003	0.997
Duration (years)	3.46 ± 1.41	3.52 ± 1.34	3.41 ± 1.47	0.084	0.919
Affected Areas				–	–
Face and neck	17	16	17		
Limbs	13	15	14		
Trunk	15	14	13		
Joints	10	10	11		
Family History				–	–
Present	9	11	10		
Absent	46	44	45		

### 3.2 Comparison of clinical treatment effects

The total effective rate of treatment in Group A was 92.73%, in Group B it was 74.55%, and in Group C it was 67.27%. The total effective rate in Group A was significantly higher than that in Groups B and C (*p* < 0.05). Refer to Table 2 for details.

**Table 2:** Comparison of clinical treatment effects.

Groups	*n*	Recovery	Significant improvement	Improvement	Ineffective	Total effective rate (%)
Group A	55	2	21	28	4	92.73%
Group B	55	1	14	27	14	74.55%^*^
Group C	55	0	8	31	18	67.27%^*^

* indicates *p* < 0.05 compared to Group A.

### 3.3 Comparison of inflammatory factor levels

**Figure 1:**
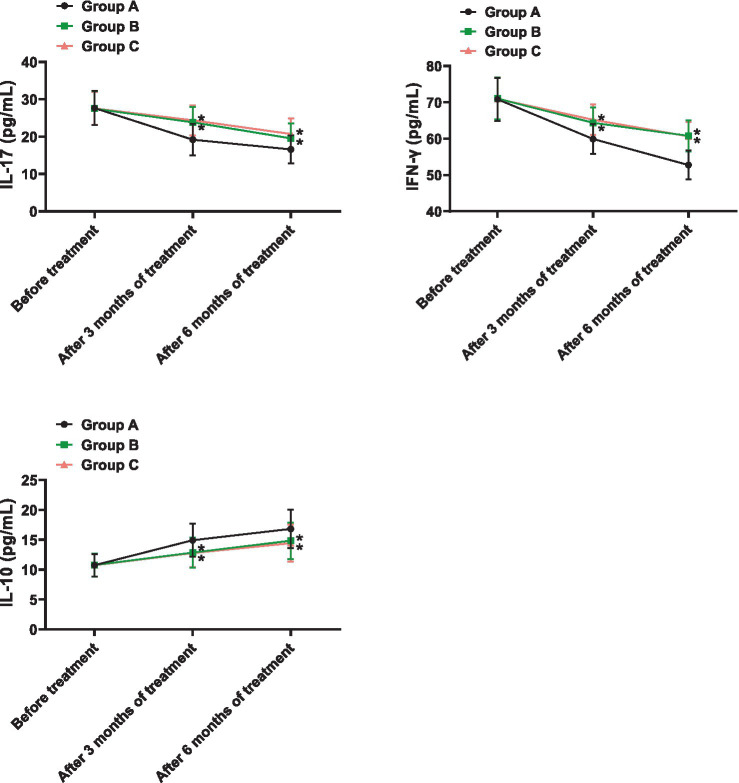
Comparison of inflammatory factor levels. * indicates *p* < 0.05 compared to Group A.

As shown in Figure 1, the levels of IL-17 in Group A before treatment and after 3 and 6 months were (27.64 ± 4.58, 19.17 ± 4.23, 16.59 ± 3.74) respectively, IFN-γ levels were (70.86 ± 5.94, 59.93 ± 4.15, 52.74 ± 3.97), and IL-10 levels were (10.74 ± 1.89, 14.93 ± 2.76, 16.82 ± 3.21). In Group B, IL-17 levels were (27.52 ± 4.63, 23.86 ± 4.11, 19.54 ± 3.97), IFN-γ levels were (71.05 ± 5.79, 64.38 ± 4.22, 60.79 ± 4.24), and IL-10 levels were (10.77 ± 1.96, 12.85 ± 2.53, 14.85 ± 3.07) before treatment and after 3 and 6 months, respectively. In Group C, IL-17 levels were (27.56 ± 4.49, 24.38 ± 4.05, 20.72 ± 4.13), IFN-γ levels were (70.95 ± 5.87, 65.23 ± 4.17, 60.64 ± 4.03), and IL-10 levels were (10.73 ± 1.92, 12.78 ± 2.36, 14.43 ± 3.11) before treatment and after 3 and 6 months, respectively. Before treatment, there were no significant differences in IL-17, IFN-γ, and IL-10 levels among the three groups (*p* > 0.05). After 3 and 6 months of treatment, the levels of IL-17 and IFN-γ decreased significantly in all three groups, while IL-10 levels increased. The extent of change in Group A was significantly higher than in Groups B and C (*p* < 0.05).

### 3.4 Comparison of prognosis in repigmentation and relapse

Regarding repigmentation, after 3 and 6 months of treatment, the repigmentation rate in Group A was significantly higher than in Groups B and C (*p* < 0.05). Concerning relapse, the relapse rate in Group A was 5.45%, in Group B it was 21.82%, and in Group C it was 23.64%. The relapse rate in Group A was significantly lower than in Groups B and C (*p* < 0.05). Refer to Table 3 for details.

**Table 3:** Comparison of prognosis in repigmentation and relapse.

Aspects	Group A (*n* = 55)	Group B (*n* = 55)	Group C (*n* = 55)	*F*	*p*
Repigmentation (3 months)	54.67 ± 2.76	46.82 ± 2.39^*^	41.47 ± 2.16^*^	404.179	<0.001
Repigmentation (6 months)	76.63 ± 2.85	59.42 ± 1.28^*^	52.83 ± 2.04^*^	1789.652	<0.001
Relapse (%)	5.45%	21.82%^*^	23.64%^*^	–	–

* indicates *p* < 0.05 compared to Group A.

### 3.5 Comparison of quality of life and psychological health

**Figure 2:**
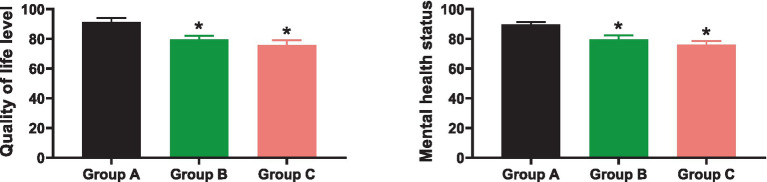
Comparison of quality of life and psychological health. * indicates *p* < 0.05 compared to Group A.

As depicted in Figure 2, at the end of the 6 month follow-up, the quality of life for patients in Groups A, B, and C was, respectively, (91.27 ± 2.68, 79.54 ± 2.51, 75.86 ± 3.23), and the psychological health condition was, respectively, (89.59 ± 1.82, 79.72 ± 2.64, 76.03 ± 2.46). The quality of life and psychological health condition in Group A were significantly higher than in Groups B and C (*p* < 0.05).

### 3.6 Comparison of adverse reaction incidence

The incidence of adverse reactions in Group A was 12.73%, in Group B was 10.91%, and in Group C was 9.09%. The comparison of adverse reaction incidence among the three groups showed no significant difference (*p* > 0.05). Refer to Table 4 for details.

**Table 4:** Comparison of adverse reaction incidence.

Adverse reaction	Group A (*n* = 55)	Group B (*n* = 55)	Group C (*n* = 55)
Fatigue	1	0	1
Burning sensation	3	2	1
Blister	2	2	0
Redness	1	2	3
Total incidence (%)	12.73%	10.91%	9.09%

* indicates *p* < 0.05 compared to Group A.

### 3.7 Histopathological and immunohistochemical analysis

Histopathological examination of skin biopsies before and after treatment revealed significant changes, particularly in Group A, compared to Groups B and C. Before treatment, histological analysis of vitiligo lesions displayed an average melanocyte count of 15 cells/mm^2^, accompanied by a moderate inflammatory infiltrate and epidermal atrophy. However, post-treatment assessment demonstrated substantial alterations in Group A, including a significant increase in melanocyte count to an average of 65 cells/mm^2^, a reduction in inflammatory infiltrate by 60%, and partial restoration of normal epidermal architecture, with a decrease in epidermal thickness by 35%, as shown in Table 5.

**Table 5:** Immunohistochemical analysis results.

Parameter	Group A	Group B	Group C
Melanocyte count (cells/mm^2^)	65 ± 10^*#^	35 ± 5^#^	40 ± 7
Epidermal thickness reduction (%)	35 ± 3^*#^	20 ± 2^#^	15 ± 2
Melan-A staining intensity (0–3+)	3 + ^*#^	2 + ^#^	2+
HMB-45 staining intensity	3 + ^*#^	2 + ^#^	2+
CD3 staining intensity reduction (%)	50 ± 5^*#^	30 ± 3^#^	25 ± 2
CD4 staining intensity reduction (%)	45 ± 4^*#^	25 ± 3^#^	20 ± 2
CD8 staining intensity reduction (%)	40 ± 3^*#^	20 ± 2^#^	15 ± 1

* indicates *p* < 0.05 when compared to Group B; # indicates *p* < 0.05 when compared to Group C.

These results indicate a marked improvement in histopathological parameters and immunohistochemical markers in Group A, supporting the observed clinical efficacy and providing insights into the underlying mechanisms of treatment response.

### 3.8 Pearson correlation analysis

Pearson correlation analysis revealed a significant positive correlation between age and treatment response (*r* = 0.35, *p* < 0.05), indicating that older age was associated with a higher likelihood of treatment response. However, there was no significant correlation observed between family history and treatment response (phi coefficient = 0.12, *p* > 0.05), suggesting that family history may not influence treatment response significantly. Similarly, gender showed no significant correlation with treatment response (phi coefficient = −0.08, *p* > 0.05), as shown in Table 6.

**Table 6:** Pearson correlation analysis.

Variable	Correlation coefficient	*p* value
Age	0.35	<0.05
Family history	0.12	>0.05
Gender	−0.08	>0.05

## 4 Discussion

Vitiligo is a common primary cutaneous disorder characterized by the loss of pigment in localized or widespread areas of the skin and mucous membranes (10). Due to its complex etiology and pathogenesis, treating vitiligo is challenging, often prone to relapse, particularly in exposed areas like the face. This significantly affects patients’ quality of life and mental well-being. Current clinical approaches to treating vitiligo primarily involve physical therapies, oral or topical corticosteroids, and surgical interventions. However, the efficacy of singular treatments tends to be low and exhibits considerable variations, often failing to achieve optimal clinical outcomes (11).

Carbon dioxide fractional laser is a minimally invasive cosmetic technique that has emerged as a means of treating vitiligo in recent years. Its mechanism involves focal photothermal action. By emitting a lattice pattern, it delivers light waves directly penetrating the dermal layer, disrupting the pigment balance between normal and vitiligo lesions. It activates dormant melanocytes or melanocyte precursors located in the outer root sheath of hair follicles within vitiligo lesions, promoting their proliferation, differentiation, and maturation into normal melanocytes that migrate to the vitiligo site (12). Moreover, it facilitates apoptosis and clearance of pathological T-lymphocytes at the lesion site, aiding repigmentation, and stimulates collagen activity, accelerating rapid wound healing (13). On the other hand, compound betamethasone injection is a corticosteroid compound that interferes with cytotoxic T-cells, reducing their cell lytic capacity. By stimulating skin follicles, it facilitates melanocyte migration, thereby regulating the restoration of pigmentation in vitiligo tissues (14). Existing clinical studies (15, 16) have demonstrated the significant therapeutic effects of both treatments for vitiligo. However, research on the combined application of these treatments and their clinical effects in vitiligo patients remains relatively scarce.

The study (17) indicates that autoimmune status, functional melanocyte loss, and genetic factors are the primary mechanisms underlying vitiligo. Recent research (18) has shown a close correlation between immune cell-mediated cytokine responses, the onset, treatment, and prognosis of vitiligo. Research (19) has found that IL-17 and IFN-γ, crucial factors secreted by Th17 and Th1 respectively, are significantly elevated in the serum of vitiligo patients, decreasing significantly after treatment. This signifies the pivotal role of IL-17 and IFN-γ in the occurrence and progression of vitiligo. Furthermore, another study (20) pointed out notable differences in the serum IL-10 levels between the general population and vitiligo patients. Variations were observed in the IL-10 levels in vitiligo patients at different stages of the condition, suggesting a close relationship between IL-10 and the disease’s activity and clinical staging. IL-10 is known to impair lymphocytes’ ability to activate Th1, decrease immune cell function, and directly counteract the proliferation of Th1 cells and the generation of factors like IL-2, IFN-γ, participating in cellular immune and inflammatory responses. This suggests IL-10’s significant role as a target for inflammation and immune suppression in vitiligo treatment (21).

In this study, compound betamethasone was used in the form of injections rather than creams or ointments to ensure deeper penetration and a more targeted delivery of the medication to the affected areas. Although compound betamethasone cream can be directly applied to the skin, injections were chosen to ensure more effective modulation of the inflammatory response and provide a controlled and sustained release of the corticosteroid. Although long-acting depot preparations like triamcinolone could also be considered for their sustained release properties, compound betamethasone was chosen due to its well-documented efficacy and safety profile in treating vitiligo. Additionally, the specific pharmacokinetics and anti-inflammatory properties of compound betamethasone make it a suitable choice for this combined treatment approach.

The parameters utilized in CO_2_ fractional laser treatment were meticulously selected, taking into account various factors such as the color and severity of vitiligo lesions, patient skin type, and desired treatment outcomes. Specifically, the superficial energy was set at 100–120 mJ/cm^2^ to ensure effective treatment of superficial pigmented lesions while mitigating damage to surrounding tissue. This energy level strikes a balance between therapeutic efficacy and safety. Additionally, the coverage was adjusted to 40% to ensure comprehensive treatment of the affected area while minimizing the likelihood of adverse effects.

In targeting deeper layers of the skin and stimulating collagen production for enhanced skin regeneration, the deep energy was set at 9–12 mJ/cm^2^. This parameter choice facilitates overall improvement in skin texture and promotes the migration and proliferation of melanocytes towards the vitiliginous lesions. Moreover, with a coverage of 5%, the treatment focuses on deeper pigmented lesions, minimizing the risk of complications such as scarring or hyperpigmentation. This approach allows for precise and targeted treatment delivery, optimizing therapeutic outcomes while ensuring patient safety and comfort.

The results of this study indicate: ① clinical treatment efficacy: Group A had a significantly higher total effective rate (92.73%) than Groups B (74.55%) and C (67.27%) (*p* < 0.05). ② Inflammatory factors: after 3 and 6 months of treatment, the average levels of IL-17 and IFN-γ decreased significantly while IL-10 levels increased significantly in all three groups. The magnitude of change in Group A was significantly higher than in Groups B and C (*p* < 0.05). ③ Prognosis in terms of repigmentation and relapse: regarding repigmentation, after 3 and 6 months of treatment, Group A showed significantly higher repigmentation rates than Groups B and C (*p* < 0.05). In terms of relapse, Group A exhibited a significantly lower relapse rate (5.45%) than Groups B (21.82%) and C (23.64%) (*p* < 0.05). ④ Quality of life and psychological health: after 6 months of follow-up, patients in Group A exhibited significantly higher levels of both quality of life and psychological health compared to Groups B and C (*p* < 0.05). ⑤ Adverse reaction incidence: the incidence of adverse reactions in Groups A, B, and C was 12.73, 10.91, and 9.09%, respectively, showing no significant difference among the three groups (*p* > 0.05). These findings suggest that the combined therapy of carbon dioxide fractional laser and compound betamethasone injection for vitiligo demonstrates better clinical outcomes and prognostic value compared to singular treatment methods. The positive correlation between age and treatment response suggests that older patients may have a better response to the treatment protocol used in this study. This finding is consistent with previous research indicating that age can influence treatment outcomes in vitiligo patients. The lack of significant correlation between family history and treatment response contradicts some previous studies, which have suggested a potential role of genetic factors in treatment response. However, it is important to consider that the sample size and specific treatment protocol used in this study may have influenced these results. The reasons behind this might involve: ① the ability of compound betamethasone to promote melanocyte generation and functionality while inhibiting their cell rupture and death (22); ② the ability of carbon dioxide fractional laser to enhance blood circulation and cellular nutritional supply at treatment sites, aiding in the recovery of various cytokine secretions and cellular immune functions, stimulating growth, proliferation, and migration of melanocyte precursors at basal layers and the periphery of vitiligo areas, facilitating melanocyte formation; ③ the complementary action of carbon dioxide fractional laser and compound betamethasone: the former primarily acts on the superficial layers of the epidermis and dermis through vaporization, followed by the use of compound betamethasone, which facilitates deeper penetration into the stratum corneum, mutually enhancing their effects. Similar results were found by Cunha and other researchers (23), confirming the efficacy of carbon dioxide fractional laser combined with compound betamethasone in improving the treatment outcomes for vitiligo patients. ④ The mechanism of action of fractional CO_2_ laser in decreasing inflammatory markers involves a multifaceted process. Initially, the laser induces controlled injury by creating microscopic columns of thermal damage in the skin while leaving surrounding tissue intact (24). This injury triggers a wound healing response characterized by the stimulation of fibroblasts to produce new collagen, promoting skin remodeling and tightening. Importantly, fractional CO_2_ laser treatment downregulates the expression of pro-inflammatory cytokines like IL-17 and IFN-γ, while promoting the release of anti-inflammatory cytokines such as IL-10 (13). Additionally, the treatment enhances skin barrier function by promoting epidermal regeneration, thereby reducing inflammation by preventing irritants and pathogens from penetrating the skin. Fractional CO_2_ laser treatment modulates the activity of immune cells in the skin, such as T cells and macrophages, resulting in a decrease in inflammatory cell infiltration and a reduction in the release of pro-inflammatory mediators (25). Overall, fractional CO_2_ laser treatment exerts its anti-inflammatory effects through tissue remodeling, cytokine modulation, and immune cell regulation, leading to an improvement in inflammatory skin conditions. ⑤ Patients undergoing combinational treatment may experience a heightened risk of adverse effects due to potential synergistic effects, increased treatment intensity, and interactions between treatments. Combining CO_2_ fractional laser with compound betamethasone injections may amplify corticosteroid-related side effects or cause unexpected reactions due to the interaction between laser-induced tissue injury and corticosteroid effects.

Moreover, histopathological examination showed significant improvements in Group A post-treatment, with a notable increase in melanocyte count (from 15 to 65 cells/mm^2^), a 60% reduction in inflammatory infiltrate, and partial restoration of epidermal architecture, resulting in a 35% decrease in epidermal thickness compared to pre-treatment levels. These results indicate a marked improvement in histopathological parameters and immunohistochemical markers in Group A, supported by significant t-values and *p*-values compared to Groups B and C. This provides insights into the underlying mechanisms of treatment response and supports the observed clinical efficacy.

## 5 Conclusion

The application of carbon dioxide fractional laser combined with compound betamethasone has shown significant effectiveness in vitiligo patients. Compared to singular treatments involving either carbon dioxide fractional laser or compound betamethasone injection, this combined approach further enhances the patients’ inflammatory factor levels, reduces the risk of recurrence, improves repigmentation, elevates their quality of life, and enhances their psychological well-being. Importantly, this combined approach does not increase the risk of adverse reactions in patients.

However, it’s crucial to note that despite the positive findings in analyzing the clinical efficacy of carbon dioxide fractional laser combined with compound betamethasone in treating vitiligo and its impact on inflammatory factors, this study has several limitations that need improvement. For instance: ① sample size and scope: the sample size in this study was relatively small and conducted only in a single medical institution, potentially affecting the stability and generalizability of the results. ② Short follow-up period: while the study included follow-ups at 3 and 6 months, considering vitiligo as a chronic condition, longer follow-up periods might better evaluate treatment outcomes and relapse scenarios. ③ Inadequate consideration of other factors: unaccounted factors like patient lifestyle, dietary habits, environmental aspects, etc., might have influenced treatment outcomes and inflammatory factor levels, which were not comprehensively controlled or considered in this study. ④ Lack of biomarker analysis: although the study involved determining inflammatory factors, it did not comprehensively explore other potential biomarkers or disease mechanisms, which could provide a more comprehensive understanding of treatment mechanisms and outcomes. ⑤ Insufficient long-term safety observation: the study did not thoroughly observe and evaluate the long-term safety of the treatment regimen. Potential risks or side effects not observed in the short term might manifest over extended use. In summary, these limitations underscore certain constraints in the study’s findings. In future research, we aim to enhance and deepen this field of study by expanding sample sizes, conducting more extended follow-ups, and addressing the mentioned limitations.

## Data availability statement

The original contributions presented in the study are included in the article/supplementary material, further inquiries can be directed to the corresponding author.

## Ethics statement

The studies involving human participants were reviewed and approved by Affiliated Hospital of Hebei Engineering University. Written informed consent to participate in this study was provided by the participants.

## Author contributions

LZ: Writing – original draft, Writing – review & editing. JZ: Writing – original draft, Writing – review & editing. XW: Writing – review & editing. ZZ: Writing – review & editing. ZL: Writing – review & editing. GM: Writing – review & editing. CL: Writing – review & editing.
